# Modulation of Metabolism and Switching to Biofilm Prevail over Exopolysaccharide Production in the Response of *Rhizobium alamii* to Cadmium

**DOI:** 10.1371/journal.pone.0026771

**Published:** 2011-11-09

**Authors:** Mathieu Schue, Agnes Fekete, Philippe Ortet, Catherine Brutesco, Thierry Heulin, Philippe Schmitt-Kopplin, Wafa Achouak, Catherine Santaella

**Affiliations:** 1 CEA, Lab Ecol Microbienne Rhizosphere & Environm Extre, iBEB, DSV, Saint-Paul-lez-Durance, France; 2 CNRS, Unite Mixte Rech Biol Vegetale & Microbiol Enviro, UMR 6191, Saint-Paul-lez-Durance, France; 3 Université Aix Marseille, Saint-Paul-lez-Durance, France; 4 Helmholtz-Zentrum Muenchen-German Research Center for Environmental Health, Institute for Ecological Chemistry, Department of BioGeochemistry and Analysis, Neuherberg, Germany; 5 Department for Chemical-Technical Analysis Research Center Weihenstephan for Brewing and Food Quality, Technische Universität München, Freising-Weihenstephan, Germany; Auburn University, United States of America

## Abstract

Heavy metals such as cadmium (Cd^2+^) affect microbial metabolic processes. Consequently, bacteria adapt by adjusting their cellular machinery. We have investigated the dose-dependent growth effects of Cd^2+^ on *Rhizobium alamii*, an exopolysaccharide (EPS)-producing bacterium that forms a biofilm on plant roots. Adsorption isotherms show that the EPS of *R. alamii* binds cadmium in competition with calcium. A metabonomics approach based on ion cyclotron resonance Fourier transform mass spectrometry has showed that cadmium alters mainly the bacterial metabolism in pathways implying sugars, purine, phosphate, calcium signalling and cell respiration. We determined the influence of EPS on the bacterium response to cadmium, using a mutant of *R. alamii* impaired in EPS production (MSΔGT). Cadmium dose-dependent effects on the bacterial growth were not significantly different between the *R. alamii* wild type (wt) and MSΔGT strains. Although cadmium did not modify the quantity of EPS isolated from *R. alamii*, it triggered the formation of biofilm *vs* planktonic cells, both by *R. alamii* wt and by MSΔGT. Thus, it appears that cadmium toxicity could be managed by switching to a biofilm way of life, rather than producing EPS. We conclude that modulations of the bacterial metabolism and switching to biofilms prevails in the adaptation of *R. alamii* to cadmium. These results are original with regard to the conventional role attributed to EPS in a biofilm matrix, and the bacterial response to cadmium.

## Introduction

The exposure of bacterial cells to heavy metals in their environment mediates biological effects, usually through the direct or indirect action of reactive oxygen species [1], [2]. In fact, non-redox-reactive metals, such as cadmium, show a high degree of reactivity towards sulfur, nitrogen and oxygen atoms in biomolecules. Cadmium may bind sulfur in essential enzymes, and alter their functions. Many studies have focused on the molecular mechanism of bacterial cell tolerance to cadmium, mainly for the case of species that are resistant to high metal concentrations, such as *Stenotrophomonas* [3] or *Cupriavidus metallidurans* (review in [4]). However, cadmium concentrations and its availability in metal contaminated soils are generally low. At low cadmium concentrations, Dedieu *et al.* [5] studied the interactions of *Sinorhizobium meliloti* extracellular compounds on cadmium speciation and availability, and Pagès et al. [6] reported on the completely different adaptation mechanisms of phenotypic variants of *Pseudomonas brassicacearum* in the presence of cadmium. Varied mechanisms account for cadmium detoxication in bacteria, involving exclusion, binding and sequestration. Cadmium is removed from cells by metal efflux transporters [7], [8], [9], reduced as cadmium sulfide [10], precipitated as insoluble salts [11], immobilized within the cell walls [12], or linked to chelating agents [13], [14]. Cell exudates, such as proteins, siderophores and to a minor extent polysaccharide, play a role in the short-term interaction between *Sinorhizobium meliloti* and cadmium [5], [15].

Because the adsorption of cadmium as well as of other metals can be associated with the secretion of exopolysaccharide (EPS) or capsular material [2], [16], [17], EPSs are considered as potential metal transporters in soil [18], [19].

Gram-negative soil bacteria belonging to the commonly named rhizobia are able to produce EPSs with a large diversity of chemical structures [20], [21]. These EPSs are the main contributors in legume-rhizobia interactions, leading to nodulation and nitrogen fixation. Eventually, much of what we know about rhizobia and their EPSs arises from studies of their symbiotic interactions with legume plants, whereas their interactions with non-legumes have been neglected. However, rhizobia associate with the roots of non-legumes such as *Arabidopsis thaliana* [22], [23], *Helianthus annuus* [24] and *Brassica napus* [25]. Rhizobial EPSs also have functions beyond specific recognition in the nodulation process, such as plant growth promotion of non-legumes [24] or evasion from the defense response of plant legumes during crack entry in roots [26]. We should consider communities of rhizobia and their EPSs as integral and functionally important partners of a diverse plant rhizosphere.

For that purpose, we addressed the question of how *Rhizobium alamii* [27], an EPS-producing bacterium, responds to the toxic impact of cadmium. *R. alamii* is a rhizobacterium, isolated from the rhizophere of the sunflower, producing a mucoid and an adhesive EPS [24]. This bacterium colonizes the root system of the sunflower, *A. thaliana*, and rapeseed, and together with its production of EPS in the rhizosphere, it improves the physical structure of the root adherent soil and plant growth under conditions of hydric stress [23], [24]. We studied the mechanisms through which EPS could contribute to the tolerance of *R. alamii* to cadmium, by using a mutant strain impaired in EPS production (MSΔGT) [23]. We investigated cadmium adsorption on the EPS, in the absence or in competition with calcium. Ion cyclotron resonance Fourier transform mass spectrometry (ICRFT/MS) was used to monitor the metabolic perturbations after exposure of *R. alamii* cells to a cadmium concentration, which slowed down, but did not inhibit cell growth. We discuss the means by which the bacterial cells adjusted both their entire metabolic processes, and their way of life, to limit cadmium damage. We have for the first time shown that cadmium promotes the formation of a biofilm by *R. alamii*, and that this change occurs independently of the presence of EPS. These results are original with regard to the conventional role attributed to EPS in a biofilm matrix, and the bacterial response to a heavy metal.

## Results and Discussion

### Effect of cadmium on *R. alamii* growth and EPS production

**Figure 1:**
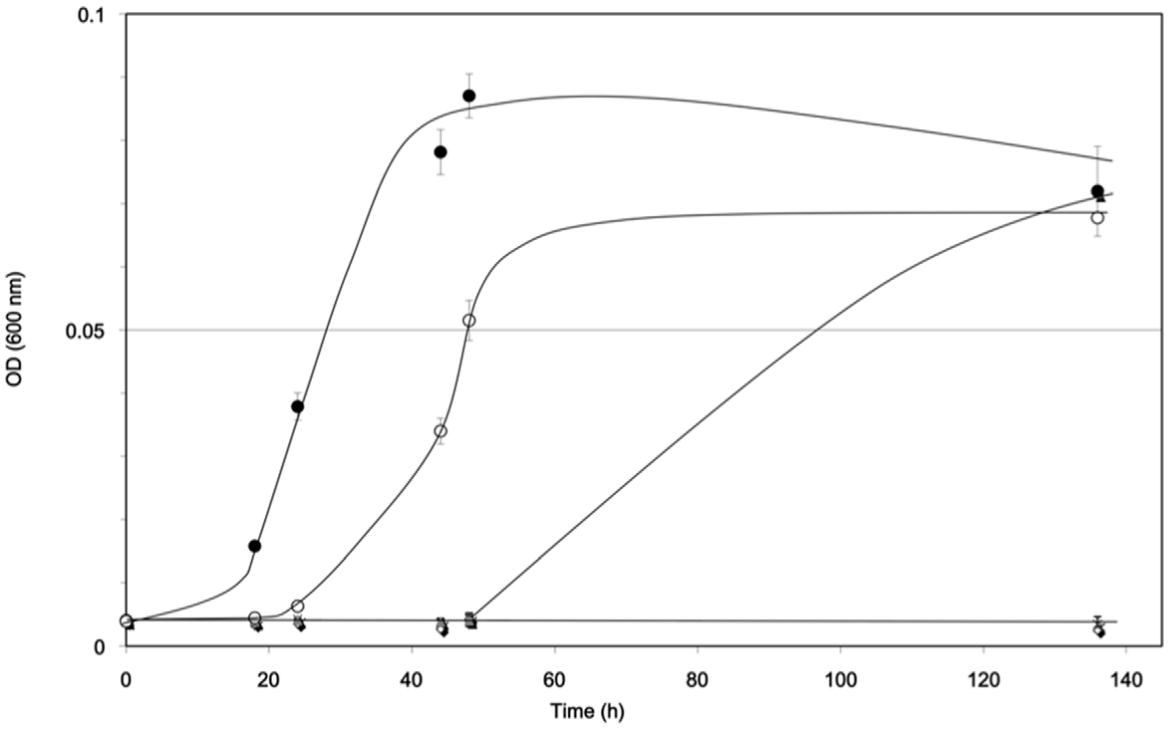
Growth curves of *Rhizobium alamii* in ten-fold diluted tryptic soy browth (TSB/10) at 30°C in the presence of cadmium nitrate (0 to 10 mg.mL-1 of Cd^2+^ (1 to 89 µM). Dark circle 0 mg.L^−1^; Open circle 1 mg.L^−1^; Grey triangle 2 mg.L^−1^ ; Cross 5 mg.L^−1^; Lozenge 10 mg.L^−1^. Mean of 3 replicates ± SD.

**Figure 2:**
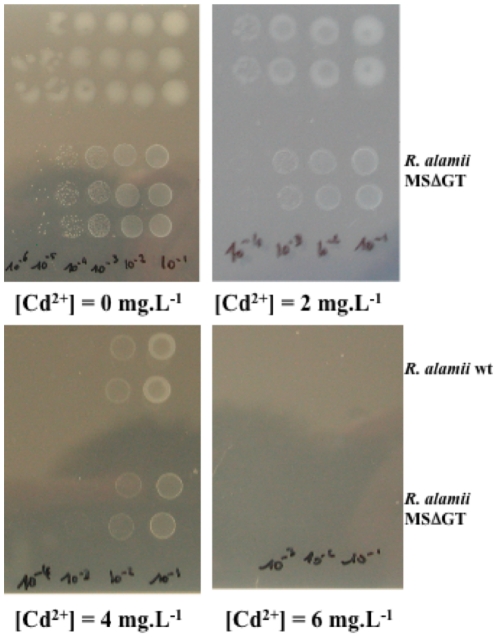
Determination of minimal inhibitory concentrations of cadmium on *R. alamii* wt and MSΔGT mutant growth on ten-fold diluted tryptic soy browth (TSB/10) solidified with agarose. Initial bacterial cell cultures in TSB/10 (10^8^ CFU.mL^−1^) were spotted at dilutions from 10^−1^ to 10^−4^. Plates were incubated at 30°C for 96 h.

In this study, the minimal inhibitory concentration (MIC) of cadmium (Cd^2+^) in *R. alamii* cells was assessed at 44 µM (5 mg.L^−1^ Cd^2+^) in a tenfold diluted Tryptic Soy Broth (TSB/10) and as illustrated in Figure 1. The increase of Cd^2+^ concentration from 1 mg.L^−1^ to 2 mg.L^−1^ almost doubled the lag phase. In our hands, cadmium MIC was 53 µM (6 mg.L^−1^) on *R. alamii* wt and MSΔGT mutant cells on an agarose solidified TSB/10 medium (Figure 2). ^1^H NMR spectra of the EPS isolated from the bacteria, cultured in the absence or presence of cadmium, showed that the chemical structure of the EPS was unmodified (A. Heyraud, personal communication). Cadmium concentrations ranging from 0 to 133 µM (0 to 15 mg.L^−1^ of Cd^2+^) did not significantly (p>0.5) modify the amount of EPS synthesized by the wt strain in a RCV mineral medium at pH 6.8, supplemented with glucose (Figure S1). The *R. alamii* behaved like *Sinorhizobium meliloti* whose EPSs content is unmodified, at pH 7 in response to 10 µM of cadmium nitrate [28].

### *R. alamii* metabonomics in response to cadmium

**Figure 3:**
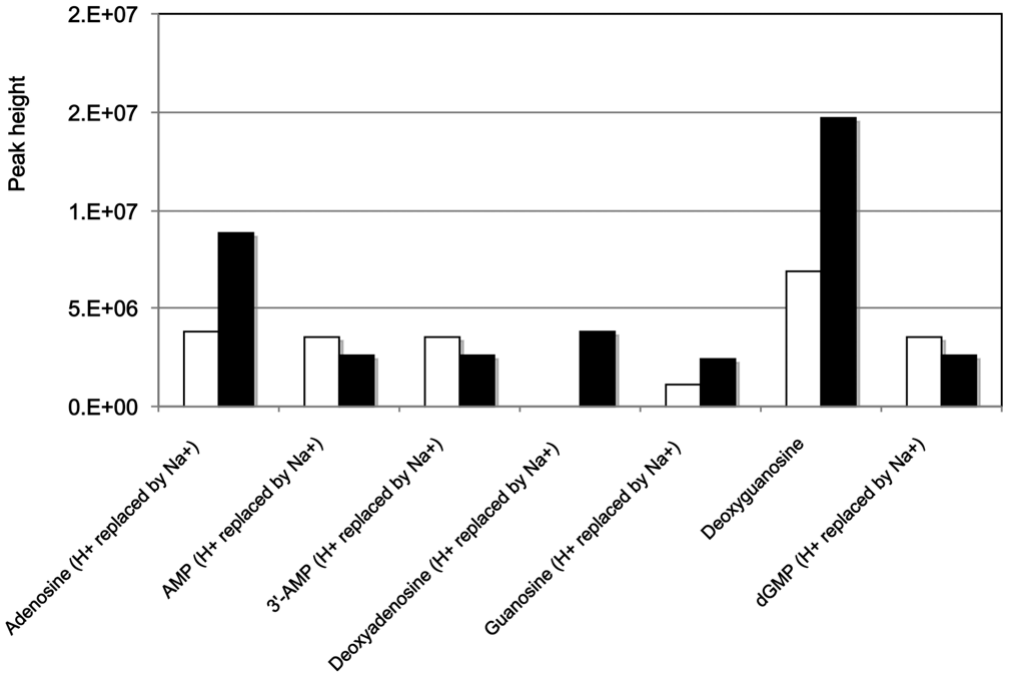
Analysis of purine and pyrimidine metabolism from *R. alamii* cells grown with no cadmium (white bars) or 2 mg.L-1 of cadmium as cadmium nitrate. Masses of the purine based cyclic nucleotides were additionally detected from the Cd^2+^ treated samples. 2,3&3,5-cyclic-AMP from the wt and 2,3&3,5-cyclic-GMP were detectable according to the FTMS measurements and use of MassTrix for data interpretation. Generally, increase in the intensity of the purine based nucleosides and decrease of the detected nucleotides were observed in presence of Cd^2+^.

**Figure 4:**
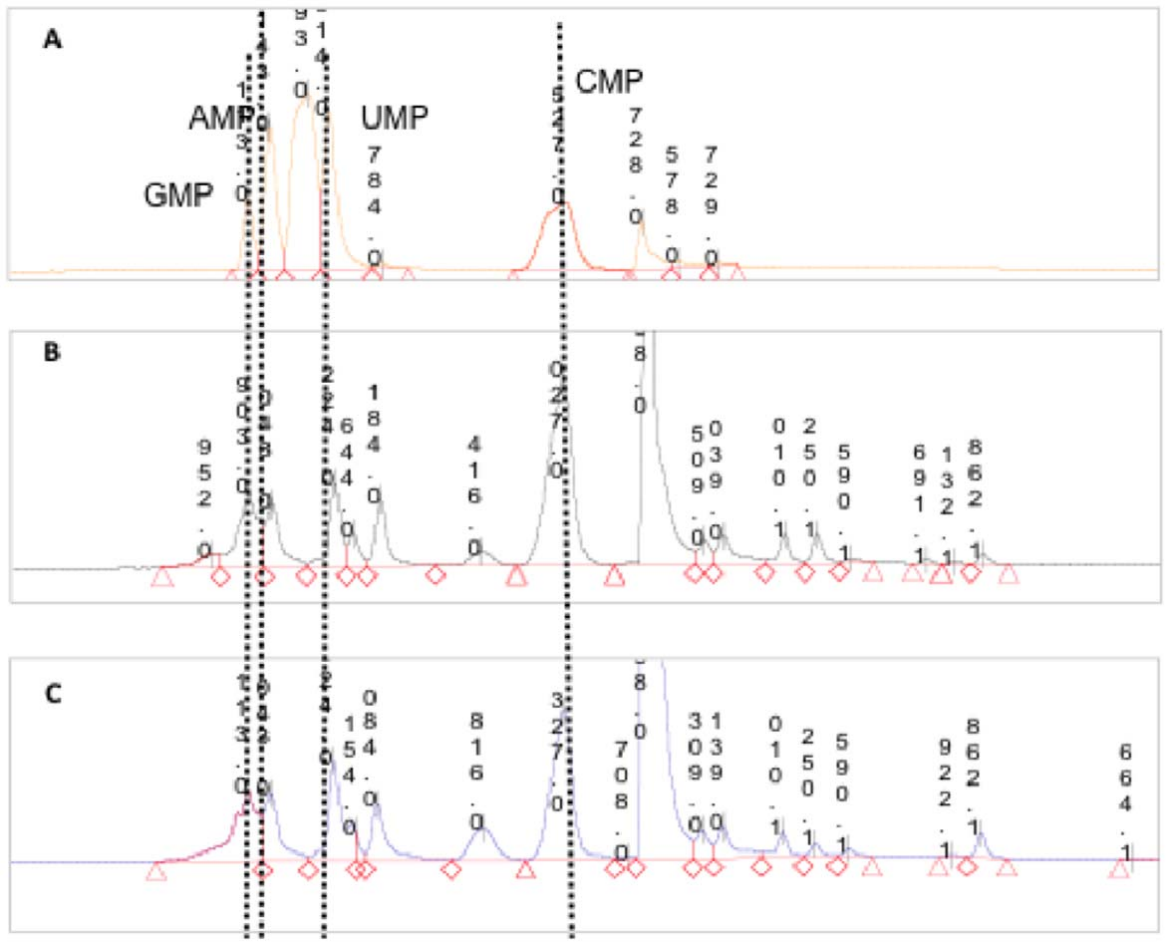
Analysis of nucleotide and nucleoside derivatives. UPLC chromatograms of cell extracts of *R. alamii* grown up to the beginning of the stationary phase. a) Blank; b) *R. alamii* cells grown in the absence of cadmium; c) *R. alamii* cells grown in the presence of 2 mg.L^−1^ of cadmium. Dash lines stand for nucleotide standards (AMP,GMP;CMP,UMP,dGTP).

We carried out metabonomic investigations on cells grown up to the end of the exponential phase, with or without 18 µM (2 mg.L^−1^) Cd^2+^, in order to determine which metabolism pathways were altered by cadmium in *R. alamii*. We used a screening method based on ion cyclotron resonance Fourier transform mass spectrometry (ICRFT/MS). This technology allows high-precision measurements to be made of a charged mass, within an error range of only a few parts per million. MassTRIX (http://masstrix.org) allows the compounds, detected with a chemically probable structure, to be assigned in the context of restricted metabolite possibilities for a given organism, using the KEGG pathway database: http://www.genome.jp/kegg/pathway.html [29]. By considering the set of *R. alamii* metabolites detected in positive and negative ESI of MS, in the experiments with or without cadmium, 1897 putative compounds were common to these conditions, and respectively 936 and 653 probable components were exclusively expressed, in the presence or absence of the metal. Cadmium induced metabolic alterations in major pathways, as summarized in Tables 1 and 2. Cadmium increased the number and level of enzymes and metabolites involved in the sugar metabolism (potentially fructose, glucose, mannose, galactose, cellobiose, inositol, starch and sucrose), phosphorylated intermediates of glycolysis compounds (likely glucose 6-phosphate, fructose 6-phosphate, glucose 1-phosphate), ABC transporters of sugars (potential methyl-galactoside, D-allose, fructose, cellobiose) and the phosphotransferase system which, in bacteria, is the major carbohydrate transport system for incoming sugar substrates, through translocations across the cell membrane. Cadmium ions are admitted into sensitive bacterial cells by the energy-dependent manganese transport systems, where they cause rapid cessation of respiration by binding to sulfhydryl groups in proteins [30], [31]. Glycolysis therefore appeared to be an alternative pathway for energy. Under cadmium stress, hexoses were potentially channelled towards a pentose phosphate pathway. In the presence of Cd^2+^, the nucleotide metabolism revealed a modulation of the purine metabolism, with an accumulation of purine-based nucleosides (adenosine, deoxyadenosine, guanine and deoxyguanine) at the same time as a decrease in purine-based nucleotides (AMP and dGMP) as illustrated in Figure 3. This result was confirmed by ultra performance liquid chromatographic (UPLC) analysis (Figure 4). The decrease in nucleotide contents and the recovery of pyrimidine and purine compounds could be consistent with an adaptation process, through slowing of cell division in cells likely to adapt to the metal toxicity [32]. In the presence of cadmium, the citric acid cycle and tryptophan metabolism were shut off (Table 1) suggesting the cessation of aerobic sugar respiration and amino acid synthesis. A glutathione precursor, **γ**-L-Glutamyl-L-cysteine, was identified under cadmium stress. In response to cadmium, *Pseudomonas brassicacearum* is found to switch from the citric acid cycle to an anaerobic metabolism [6], and the inhibition of protein and glutathione syntheses are described in the response of *R. leguminosarum* to cadmium [14], [33]. Cadmium activated the biosynthesis of lipopolysaccharide and capsular polysaccharide, which are the outer membrane components of Gram-negative bacteria and are potential metal binding sites [6], [12], [34]. Pyrophosphate was detected following Cd^2+^ treatment only. Pyrophosphate can form crystals with cadmium [35]. The accumulation of inorganic phosphate is a detoxification mechanism reported in *Klebsiella aerogenes* [36] and is also involved in the regulation of biofilm formation in *Pseudomonas fluorescens* [37]. Cadmium is also activated the inositol phosphate metabolism that regulates cytoplasmic Ca^2+^ and communication, and the cyclic ADP-ribose pathway, which is a secondary messenger for Ca^2+^ mobilisation [38]. The structural homology between calcium and cadmium ions might account for the activation of pathways needed to maintain calcium homeostasis and signal transduction.

**Table 1:** Modification of KEGG pathway metabolites induced by cadmium in *Rhizobium alamii*.

KEGG pathway	Number of identified metabolitesCadmium concentration (mg.L^−1^)	
	0	2
**Glycolysis/Gluconeogenesis**	0	7
**Citrate cycle (TCA cycle)**	3	0
**Pentose phosphate pathway**	0	6
**Pentose and glucuronate interconversions**	1	4
**Fructose, mannose, inositol and galactose metabolism**	12	44
**Purine, pyrimidine metabolism**	30	26
**Lysine biosynthesis**	10	2
**Glutathione metabolism**	1	1
**Starch and sucrose metabolism**	6	21
**Nucleotide sugars metabolism**	0	2
**Lipopolysaccharide biosynthesis**	0	1
**Peptidoglycan biosynthesis**	1	2
**Inositol phosphate metabolism**	0	7
**ABC transporters**	13	20
**Two-component systems**	0	2
**Phosphotransferase system**	9	17

*Rhizobium alamii* cells were grown up to a late exponential phase, with 2 mg.L^−1^ of cadmium, as compared to the absence of cadmium. The potential metabolites identified were matched in KEGG pathway using MassTRIX (http://masstrix.org).

**Table 2:** Main hypotheses in the metabolic pathways altered by cadmium.

Activation of pathways	Hypothesis
Sugar metabolism	The cessation of respiration by binding of cadmium to
ABC transporters of sugars	sulfhydryl groups in proteins induces the glycolysis or
Phosphorylated intermediates of glycolysis	the shift towards the pentose phosphate pathway as an
Phosphotransferase system	alternative pathway for energy
Pentose phosphate pathway	
Biosynthesis of lipopolysaccharide and capsular polysaccharide	Outer membrane components of Gram-negative bacteria are metal binding sites
Pyrophosphate	Cadmium binding to pyrophosphate
Inositol phosphate metabolism	Regulation of cytoplasmic Ca^2+^ and communication
γ-L-Glutamyl-L-cysteine	Glutathione precursor

Inhibition or decrease in pathways	Hypothesis
Citric acid cycle	The cessation of aerobic sugar respiration and amino
Tryptophan metabolism	acid synthesis
Nucleotides	Cell division is slowed to accommodate to the toxicity

KEGG pathways activated or inhibited in the response of *Rhizobium alamii* to cadmium (2 mg.L^−1^).

Altogether, these results show that the adaptation of *R. alamii* cells to cadmium could imply a multifaceted scheme using metal binding, precipitation or export, changes in respiration process, and the retention of cadmium-like metal homeostasis.

### Cadmium induces the formation of a biofilm by *R. alamii* independently of EPS synthesis

**Figure 5:**
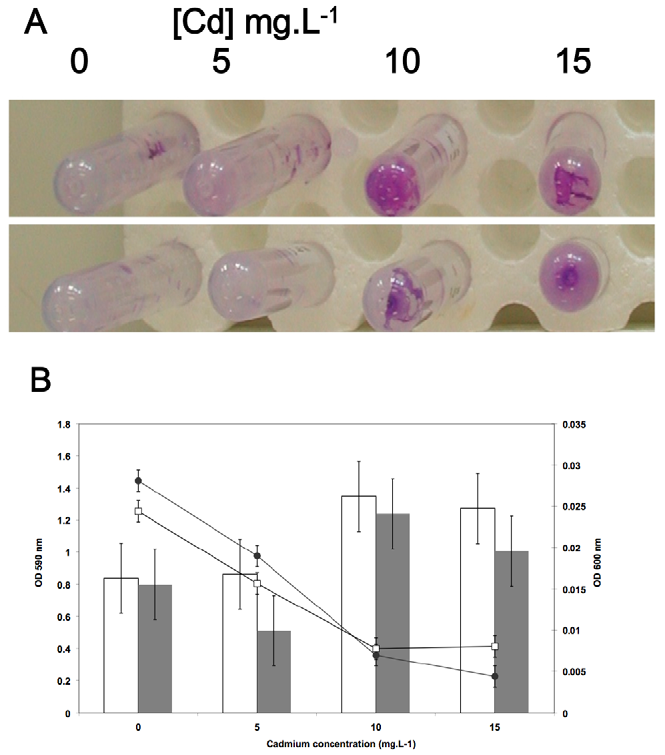
Enhancement of biofilm formation in response to cadmium. A) Staining of *Rhizobium alamii* biofilms formed by the wild type strain (wt, upper panel) and the MSΔGT mutant impaired in EPS synthesis (lower panel), using a crystal violet assay (O'Toole et al, 1990). The cadmium concentration increases from left to right (0 to 133 µM; 0 to 15 mg.L^−1^). B) Biofilms of *R. alamii* wt (white bars) and MSΔGT mutant (dark bars) were quantified using a crystal violet assay (OD 595 nm) after 72 h of static culture at 30°C. Planktonic growth of *R. alamii* wt (open squares) and MSΔGT mutant (black circles) was measured in the culture supernatant (OD 600 nm). The error bars represent the confidence intervals (95%) of triplicates.

*R. alamii* wt and MSΔGT strains were grown statically in the presence of cadmium (from 0 to 15 mg.L^−1^ of Cd^2+^), in mineral RCV medium supplemented with glucose (for composition see materials and methods) to promote EPS synthesis. Figure 5A shows staining of biofilms formed on the tube walls using crystal violet [39], and Figure 5B represents the quantity of planktonic cells monitored by means of optical density measurements, and the quantification of surface-attached cells stained by crystal violet coloration as a function of cadmium concentration. Increasing cadmium concentrations significantly decreased the population of the planktonic cells, and induced the attachment of cells as a biofilm (p<0.05).

A biofilm is a lifestyle able to resist various environmental stressors such as antibiotics [40], or metals such as copper, zinc and lead [41], and nickel [42]. In the present study, we reveal for the first time cadmium-induced biofilm formation by *R. alamii* cells. Interestingly, wt and EPS-mutant strains showed the same change in growth, from free-swimming to biofilm mode, in response to cadmium, suggesting that biofilm formation, rather than EPS, was a remediation to metal toxicity.

### Cadmium binds to the EPS of *R. alamii*

The chemical structure of *R. alamii* EPS contains carboxylic and hydroxyl functions [43] that may bind metals. The biosorption of metal ions on the EPS of *R. alamii* has been measured in a MOPS buffer and in a calcium-containing medium, and has been described using the Langmuir and Freundlich isotherms [44]. The parameters calculated from these two models are summarized in Table 3. The Langmuir model, based on the assumption of a monolayer adsorption onto a solid surface with a defined number of identical sites, gave the best fit of equilibrium adsorption data measured in a MOPS buffer. The Freundlich isotherm, which is an empirical model used to describe heterogeneous systems, best fitted the biosorption of cadmium in the presence of an excess of calcium ions. Both models showed that the EPS of *R. alamii* actually bound cadmium. An apparent distribution coefficient of 3480 L.mg^−1^ was found at an EPS concentration of 0.125 g.L^−1^ and a maximum biosorption capacity of 11 mg of cadmium per g of EPS was determined. Both models showed that calcium, an abundant ion in soil solutions, competed with cadmium and reduced its sorption. At the highest EPS concentration tested (1 g.mL^−1^), the EPS of *R. alamii* showed the lowest ability to bind Cd^2+^ (apparent distribution coefficient of 387 L.mg^−1^ and maximum biosorption capacity of 6 mg of cadmium per g of EPS), showing that polysaccharide chain-chain interactions could preclude the binding of cadmium.

**Table 3:** Isotherm parameters.

*R. alamii* EPS % w/v	Medium	Langmuir Model			Freundlich model		
		Qm (mg/g)	K_L_ (L/mg)	r^2^_L_	n	K_F_ [(mg/g)(L/mg)^1/n^]	r^2^_F_
0.0125	MOPS	11 10^−3^	3 480	0.997	1.01	2.32	0.987
	Calcium nitrate	4 10^−5^	47 686	0.680	2.00	1.03	0.991
0.1	MOPS	6 10^−3^	387	0.999	1.13	0.60	0.987
	Calcium nitrate	3 10^−4^	4 976	0.983	1.14	0.37	0.987

Isotherms parameters were determined from Langmuir and Freundlich models for Cd^2+^ adsorption on the EPS of *Rhizobium alamii* in a MOPS buffer or calcium nitrate (10^−2^ M) at pH 6.5. The reaction was maintained at 25°C under stirring, pH 6.5±0.2 for 24 h and 48 h to ensure that the equilibrium was reached.

The metal uptake, X (mol.kg^−1^) is determined using: X = V. (C_0_−C_e_). M^−1^ where C_0_ and C_e_ are the concentrations of initial and equilibrium cadmium in the solution (mol.L^−1^), respectively; V is the solution volume (L); M is the mass of sorbent (kg). The error bars represent the confidence intervals (95%) of triplicates.

### Cadmium and biofilm matrix imaging by confocal laser scanning microscopy

**Figure 6:**
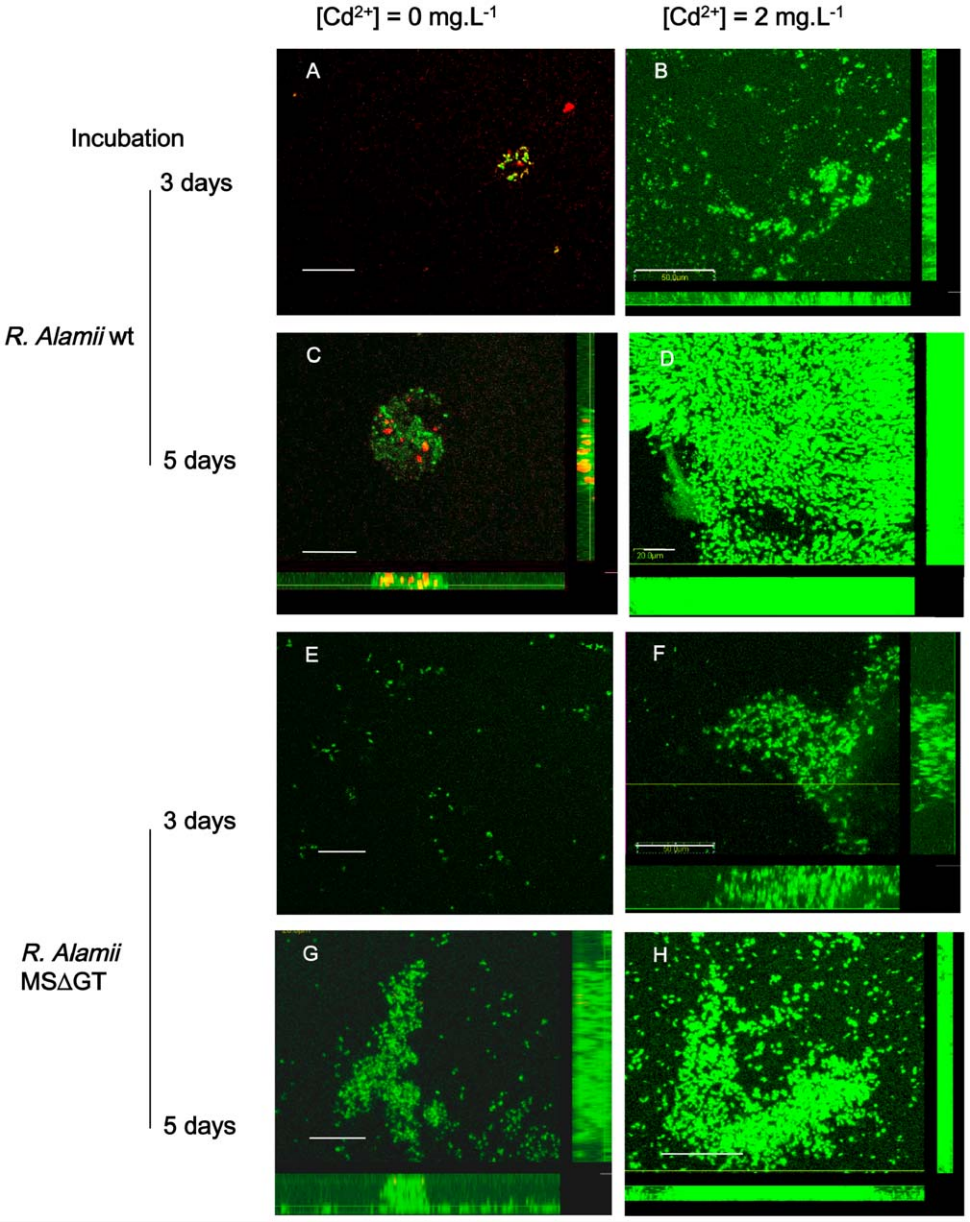
Confocal laser-scanning micrographs of *Rhizobium alamii* wt and MSΔGT mutant. mg.L^−1^ of Cd^2+^ in the form of cadmium nitrate, 3 days (left lane) and 5 days (right lane), Bacteria were grown on polycarbonate membranes in a minimal M9 medium supplemented with 2 g.L^−1^ of glucose, with 0 or 2 mg.L^−1^ of cadmium following inoculation of the medium with 2.10^5^ cell.mL^−1^. The bacterial cells were localized by constitutive expression of GFP (in green); EPS was labeled by binding with fluorescent Alexa660-Concanavalin A (in red) in observations performed at 3 days of growth. 5-day biofilms were unlabeled for EPS. A) *R. alamii* wt after 3 days of growth in the absence of cadmium nitrate. Projections of Z-sections (1 µm) through 5 µm. The scale bar corresponds to 20 µm. Few cells were attached to the membrane surface as a small core. The EPS can be seen in red, surrounding a single cell. B) After 5 days of growth in the absence of cadmium. Projections of Z-sections (1 µm) through 5 µm. The scale bar corresponds to 50 µm. Bacterial cells colonized the membrane, in the form of a loose spherical biofilm. The EPS can be seen in red in some areas of the biofilm (see picture insert projection of Z-sections through 15 µm, the scale bar corresponds to 20 µm). C) *R. alamii* wt after 3 days of growth in the presence of cadmium nitrate (Cd^2+^ 2 mg.L^−1^). Projections of Z-sections (1 µm) through 5 µm. The scale bar corresponds to 20 µm. The cells grew as a sphere. The EPS was localized inside the biofilm. D) *R. alamii* wt after 5 days of growth in the presence of cadmium nitrate (Cd2+ 2 mg.L^−1^). Projections of Z-sections (1 µm) through 15 µm. The scale bar corresponds to 20 µm. The cells were organized in the form of fused, expanded spheres. E) MSΔGT mutant cell development on a membrane surface after 3 days of growth, in the absence of cadmium nitrate. Projection of Z-sections (1 µm) through 6 µm. The scale corresponds to 20 µm. The cells adhered to the membrane surface, although as single cells. F) MSΔGT mutant after 5 days of growth in the absence of cadmium. Projections of Z-sections (1 µm) through 17 µm. The scale bar corresponds to 50 µm. The bacterial cells formed a flat, loose biofilm architecture. G) MSΔGT mutant after 3 days of growth in the presence of cadmium nitrate (Cd^2+^ 2 mg.L^−1^). Projection of Z-sections (1 µm) through 5 µm. The scale bar corresponds to 50 µm. Biofilms developed in the yz plane. 2 h. MSΔGT mutant after 5 days of growth in the presence of cadmium nitrate (Cd^2+^ 2 mg.L^−1^). Projection of Z-sections (1 µm) through 17 µm. The scale bar corresponds to 50 µm. The cells formed a distributed, dense biofilm.

In Figure 6, the structure of cell organization, single cells, microcolonies and biofilms, from *R. alamii* wt and the MS**Δ**GT mutant, were examined after 3 days and 5 days of incubation at 30°C. The constitutive expression of GFP allowed the bacterial cells to be imaged. EPS was labelled with a fluorescent lectin (left lane only). Figure 6 shows typical pictures of the development of *R. alamii* wt and MSΔGT mutant cells on a membrane, in the presence (2 mg.L^−1^) or absence of cadmium. At 3 days of growth in the absence of cadmium, the *R. alamii* wt and MSΔGT mutant colonized the surface, in the form of microcolonies (Figure 6A), or scattered single cells (Figure 6E), respectively. After 5 days of growth, *R. alamii* wt and MSΔGT formed spread biofilms with a loose architecture (Figure 6B and 6F). However, cadmium already induced the formation of condensed biofilms of both strains, after 3 days of incubation (Figs. 6C and 6G). After 3 and 5 days, in the presence of cadmium, EPS-producing *R. alamii* wt cells formed globular biofilms expanded in the z direction (Fig. 6D), whereas the MSΔGT mutant formed broad, flat and dense biofilms (Figure 6H). We conclude that the EPS production of *R. alamii* enabled the spatial propagation of the cells, and the construction of a 3D-spread biofilm matrix. This observation corroborates the contribution of bacterial exopolymers to the biofilm architecture [45], [46]. The localization of EPS in *R. alamii* biofilms grown in the presence of cadmium did not occur more frequently than in the absence of the metal, which confirms that *R. alamii* does not modulate EPS production in response to cadmium toxicity.

The view of the role of rhizobia and their EPS being restricted mainly to symbiotic interactions with legumes is insufficient to gain insight into their physiology, and ability to adapt to environmental fluctuations. Altogether, our data has revealed some unique results, related to the metabolic response and lifestyle of *R. alamii*, and to the function of the EPS in response to cadmium. The belief that EPSs are the main players in metal tolerance of bacteria is persistent in microbiology, mainly in environmental science literature. Our experiments show that the bacterial polysaccharide (EPS) of *R. alamii* can bind cadmium, although this is not the way that these bacteria use to adapt to the metal toxicity. Changes of lifestyle, from planktonic to biofilm growth, and altering the metabolism, are the means to escape metal toxicity by wt cells as well as by mutant cells that no longer produce EPS. Further studies are being designed to determine the role of *R. alamii* and its EPS on plant cadmium uptake in the rhizosphere.

## Materials and Methods

### Bacterial strains and plasmids

*Rhizobium alamii* wt (described in [24] and avalaible at the Institut Pasteur CNCM I-1809P) and MSΔGT (described in [23] and available upon request), were grown at 30°C in a tenfold diluted tryptic soy broth (DIFCO Laboratories, Detroit, USA), or in a mineral RCV medium [23], at pH 6.8, supplemented with glucose (2 g.L^−1^) as a carbon source to favour EPS synthesis or in a minimal M9 medium (NaH_2_PO_4_ 6 g.L^−1^, KH_2_PO_4_ 3 g.L^−1^, NH_4_Cl 1 g.L^−1^, NaCl 0.5 g.L^−1^, MgSO_4_ 1 mM, Thiamine 50 mg.L^−1^, L-Leucine 0.25 g.L^−1^, L-Proline 0.25 g.L^−1^, and glucose 2 g.L^−1^. Nalidixic acid, kanamycin and tetracycline were respectively used at 50 µg.mL^−1^, 25 µg.mL^−1^ and 15 µg.mL^−1^ for the appropriate antibiotic selection of *R. alamii* strains.

*Rhizobium alamii* wild type and its EPS-deficient mutant MSΔGT labelled with green-fluorescent protein (GFP) [23], [47] constitutively expressed the GFP for almost three months in soil, even in the absence of the antibiotic selection pressure.

### Cadmium adsorption isotherms

EPS from *R. alamii* was isolated as described in [23], dialyzed and lyophilized prior to use. Cadmium binding to EPS of *R. alamii* was performed as described by Nelson *et al.* [48]. EPS solutions (0.5 to 4 g.L^−1^, 10 mL) in a MOPS buffer (10^−2^ M), or CaNO_3_ (10^−2^ M), were placed inside dialysis tubing (Cellu-Sep® T3 Membranes, 12-14000 MWCO, Interchim, Montluçon, France) and equilibrated in 10 mL CdNO_3_ solutions (10^−4^ to 10^−11^ M) doped with ^109^CdCl_2_ (830 cpm.mL^−1^, 0.62 nCi final concentration). The pH was adjusted to 6.5±0.2, with NaOH 1N or HCl 1N. Triplicate samples were placed on a rotator at 22°C. Dialysis tubings with no EPS in solution were taken as blanks. Aliquots of the external solutions were taken at 24 and 48 h, and the γ-emissions were measured with a gamma counter.

### Tolerance of *Rhizobium alamii* to cadmium

The wt and MSΔGT strains were grown in a ten-fold diluted tryptic soy broth (TSB/10), or in a RCV supplemented with glucose (2 g.L^−1^) and various concentrations of cadmium (0 to 133.4 µM; 0 to 15 mg.L^−1^ of Cd^2+^). The OD_630 nm_ was measured over time from triplicate samples and reflected planktonic cell concentrations.

The *R. alamii* wt cells were grown up to an early stationary phase in TSB/10 supplemented with 0 or 2 mg.L^−1^ of cadmium nitrate. The cells were collected at OD_600 nm_ near to 0.07. Cytosolic cellular extracts were extracted in 50/50 methanol/water, in an ultrasonic bath for 15 min. The pellets were centrifuged at 10000 rpm for 15 min, and the supernatant was analyzed using a Bruker-Daltonics APEXQ 12 Tesla ICR-FT mass spectrometer (Bremen, Germany). The samples were introduced by macrospray infusion, at a flow rate of 120 µL.h^−1^, ionised in negative and positive electrosprays (ESI), and 256 scans were accumulated over a broadband range of masses (m/z 150–2000). The instrument was externally calibrated on clusters of arginine every measurement day, and the mass spectra were internally calibrated with phtalate diesters in positive ESI, and with fatty acids in negative ESI, thus ensuring a maximum error of 100 ppb. For the ICR-FT MS experiments, three replicates were analyzed for each condition (Figure S2), and the masses common to all three measurements were selected in order to generate a list of potential compounds. From the full set of detected compounds, we focused on those having a suggested attribution for the chemical structure with a difference between the detected and calculated masses of less than ±3 ppm, with detected peaks preferentially confirmed by the presence of ^18^0, ^15^N or ^13^C isotopes. The peaks exceeding a threshold signal-to-noise ratio of 3 were exported to peak lists, and were submitted to a metabolite-annotation web interface, MassTRIX (http://masstrix.org). MassTRIX processes the submitted mass peak list by comparing the input experimental masses with all of the compounds recorded in the Kyoto Encyclopedia Genes and Genome (KEGG) chemical compound database, using *Rhizobium leguminosarum* as the model organism. The MassTRIX annotation of metabolites was used to highlight differences in metabolic pathways between the *R. alamii* cells incubated with or without Cd^2+^ 2 mg.L^−1^. Moreover, the compounds identified by ICR-FT/MS were matched with the KEGG reaction components. A full table of metabolites was added as supporting information Tables S1, S2, S3, S4. The raw data and the position of the potential metabolites in KEGG pathways can be consulted at http://metabolomics.helmholtz-muenchen.de/masstrix2, section “job status”, job access numbers 10031710000016906 and 10031709591316561 for wild type cells [Cd^2+^] 2 or 0 mg/L in negative mode, and 10031709545915503 and 10031709560715818, for wt cells [Cd^2+^] 2 or 0 mg/L in positive mode.

The analyses of pyrimidine and purine metabolites, with or without cadmium culture conditions, were performed by ULPC-MS on three independent replicates, which were characterised by similar profiles, indicating a high reproducibility of the three experiments (Figure S3 and Figure S4).

### Adhesion of bacterial cells

The *R. alamii* wt and MSΔGT mutant were statically grown to a stationary phase, in polypropylene tubes containing RCV-medium, supplemented with 2 g.L^−1^ glucose and cadmium nitrate (0 to 133.4 µM; 0 to 15 mg.L^−1^ of Cd^2+^). Bacterial adhesion to solid surfaces and the formation of biofilms were monitored using a crystal violet staining-based protocol adapted from [39]. Breafly, the planktonic bacteria were removed from the tubes. Washing was performed with sterile water. After careful remove of the water, staining was performed with 2 mL of 1% crystal violet solution, 15 min at room temperature. The crystal violet solution was gently removed and successive washings were performed with water. Each tube was inverted and gently tapped on paper towels to remove any excess liquid and allow tubes to air-dry. 2 mL of 100% ethanol was added to each tube and OD_595_ was measured in a cuvette on a spectrophotometer.

### Microscopy

Biofilms of *R. alamii* wt and MSΔGT mutant were statically grown on a polycarbonate membrane (Millipore 0.2 µm) incubated in a modified M9 medium (Na_2_HPO_4_ 6 g.L^−1^, KH_2_PO_4_ 3 g.L^−1^, NH^4^Cl 1 g.L^−1^, NaCl 0.5 g.L^−1^, MgSO_4_ 1 mM, thiamine 50 mg.L^−1^, L-Leucine 0.25 g.L^−1^, L-Proline 0.25 g.L^−1^ and glucose 20 g.L^−1^), supplemented with 0 or 2 mg.mL^−1^ of cadmium nitrate. 30 mL of M9 medium were inoculated with 2.10^5^ cells of *R. alamii* wt or MSΔGT mutant, grown to half of the exponential phase in TSB/10, and washed with sterile ultrapure water. EPS labelling with concanavalinA and confocal laser scanning microscopy were performed on an Olympus confocal microscope, as described in Santaella *et al.* [23].

### Data treatment

Statistical analyses (ANOVA) were made using version XV of the Statgraphics Centrion software.

## Supporting Information

Figure S1 **Amount of EPS isolated from 5-day old R.** ***alamii*** **cultures (5 mL) in RCV medium (white bars) and in M9 modified medium (grey bars) supplemented with glucose (2 g.L^−1^). Mean of 3 replicates ± SD.**
(DOC)

Figure S2 **Examples of off-line FTMS measurements.**
(DOC)

Figure S3 **Examples of UPLC chromatographs of cell extracts of** ***R. alamii*** **grown without cadmium to the beginning of the stationary phase.UPLC-PDA, HSS column.** Eluent: A: 10 mM ammonium acetate pH 6.4, B: 20% 10 mM ammonium acetate in acetonitrile. Flow: 0.9 mL.min^−1^ (pressure drop 600–900 bar). Column temperature: 40°C. Injection: 5 µL, partial loop with needle overfill. Wavelength detection 256 nm. Illustration of the reproducibilty of the analyses from three repetitions of independent cell cultures and extracts.
(DOC)

Figure S4 **Example of UPLC chromatographs of cell extracts of** ***R. alamii*** **grown with 2 mg.L^−1^ of cadmium to the beginning of the stationary phase.** Analysis of three independent cell cultures and extracts.
(DOC)

Table S1 **Potential metabolites detected by ICR-FT MS negative ESI in** ***R. alamii*** **grown with no cadmium nitrate.**
(XLS)

Table S2 **Potential metabolites detected by ICR-FT MS positive ESI in** ***R. alamii*** **grown with no cadmium nitrate.**
(XLS)

Table S3 **Potential metabolites detected by ICR-FT MS negative ESI in** ***R. alamii*** **grown with 2 mg.L^−1^ of cadmium nitrate.**
(XLS)

Table S4 **Potential metabolites detected by ICR-FT MS positive ESI in** ***R. alamii*** **grown with 2 mg.L^−1^ of cadmium nitrate.**
(XLS)
