# Supplementary material for: Modulation of Metabolism and Switching to Biofilm Prevail over Exopolysaccharide Production in the Response of Rhizobium alamii to Cadmium
Source: PLoS One. 2011 Nov 9;6(11):e26771. doi: 10.1371/journal.pone.0026771 (PMC3212527; doi:10.1371/journal.pone.0026771)
Supplement: Supplementary file 1 [file pone.0026771.s001.doc]

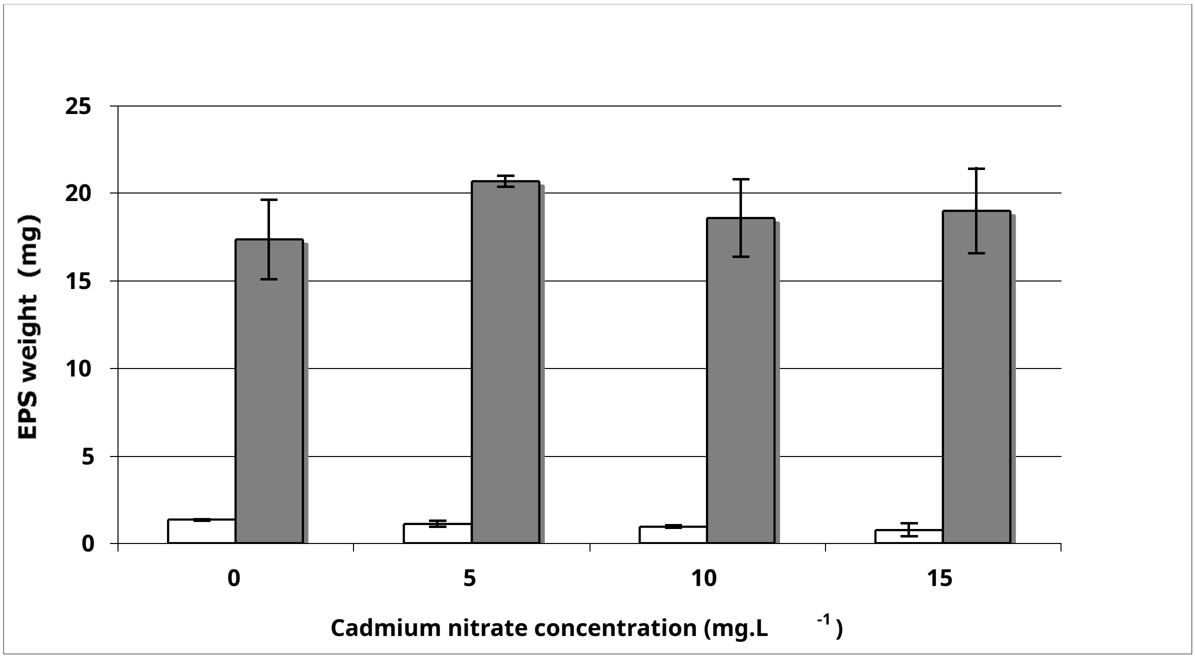

EPS weight (mg)
25
20
15
10
5
0
0
5
10
15
Cadmium nitrate concentration (mg.L-1)
