# Supplementary material for: Modulation of Metabolism and Switching to Biofilm Prevail over Exopolysaccharide Production in the Response of Rhizobium alamii to Cadmium
Source: PLoS One. 2011 Nov 9;6(11):e26771. doi: 10.1371/journal.pone.0026771 (PMC3212527; doi:10.1371/journal.pone.0026771)
Supplement: Supplementary file 2 [file pone.0026771.s002.doc]

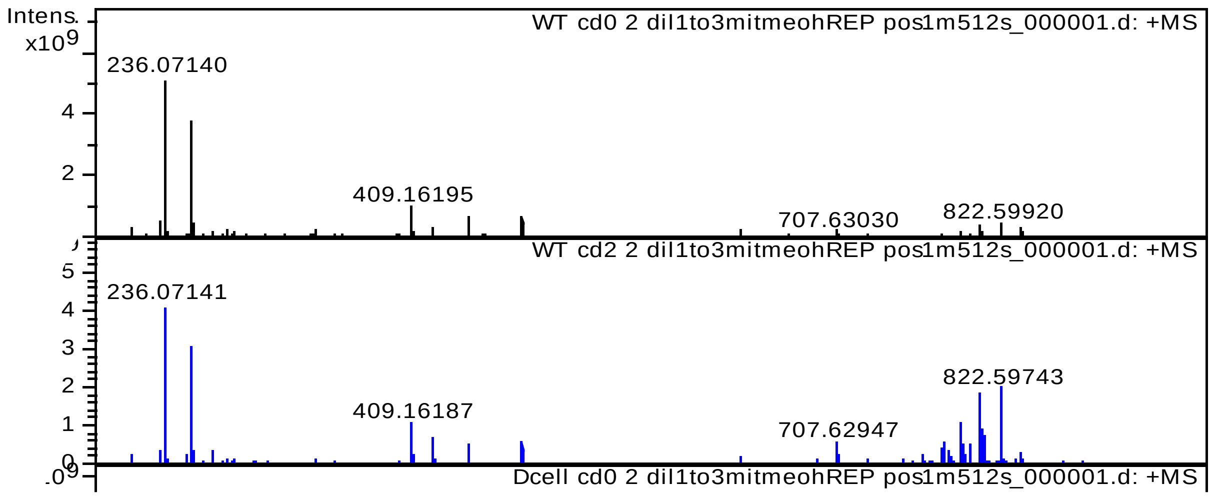
Intens.
x10^9
WT cd0 2 dil1to3mitmeohREP pos1m512s_000001.d: +MS
236.07140
409.16195
707.63030
822.59920
WT cd2 2 dil1to3mitmeohREP pos1m512s_000001.d: +MS
236.07141
409.16187
707.62947
822.59743
Dcell cd0 2 dil1to3mitmeohREP pos1m512s_000001.d: +MS
