# Supplementary material for: Modulation of Metabolism and Switching to Biofilm Prevail over Exopolysaccharide Production in the Response of Rhizobium alamii to Cadmium
Source: PLoS One. 2011 Nov 9;6(11):e26771. doi: 10.1371/journal.pone.0026771 (PMC3212527; doi:10.1371/journal.pone.0026771)

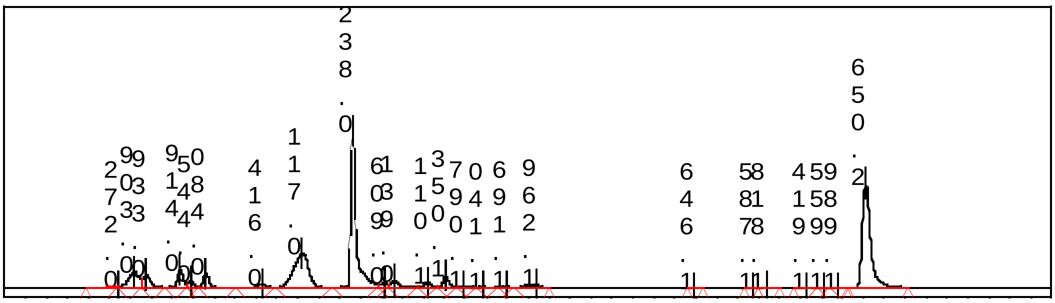

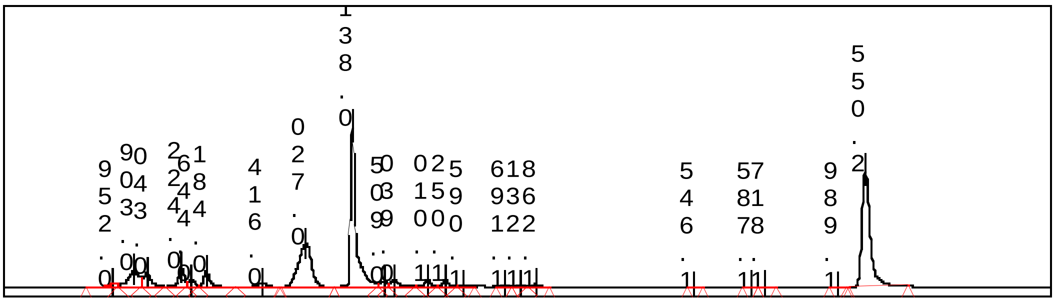

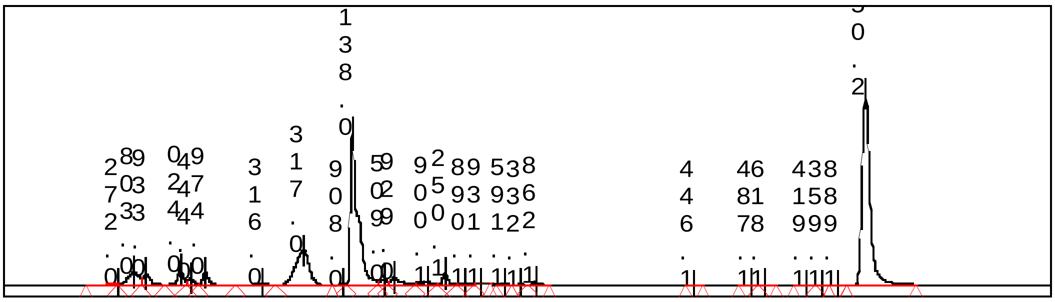

Supplement: Supplementary file 3 [file pone.0026771.s003.doc]
